# Supplementary material for: Knockout Mutants of OsPUB7 Generated Using CRISPR/Cas9 Revealed Abiotic Stress Tolerance in Rice
Source: Int J Mol Sci. 2023 Mar 10;24(6):5338. doi: 10.3390/ijms24065338 (PMC10048836; doi:10.3390/ijms24065338)
Supplement: Supplementary file 1 [file ijms-24-05338-s001.zip › (2023-2-14) Supplementary figure.pdf]

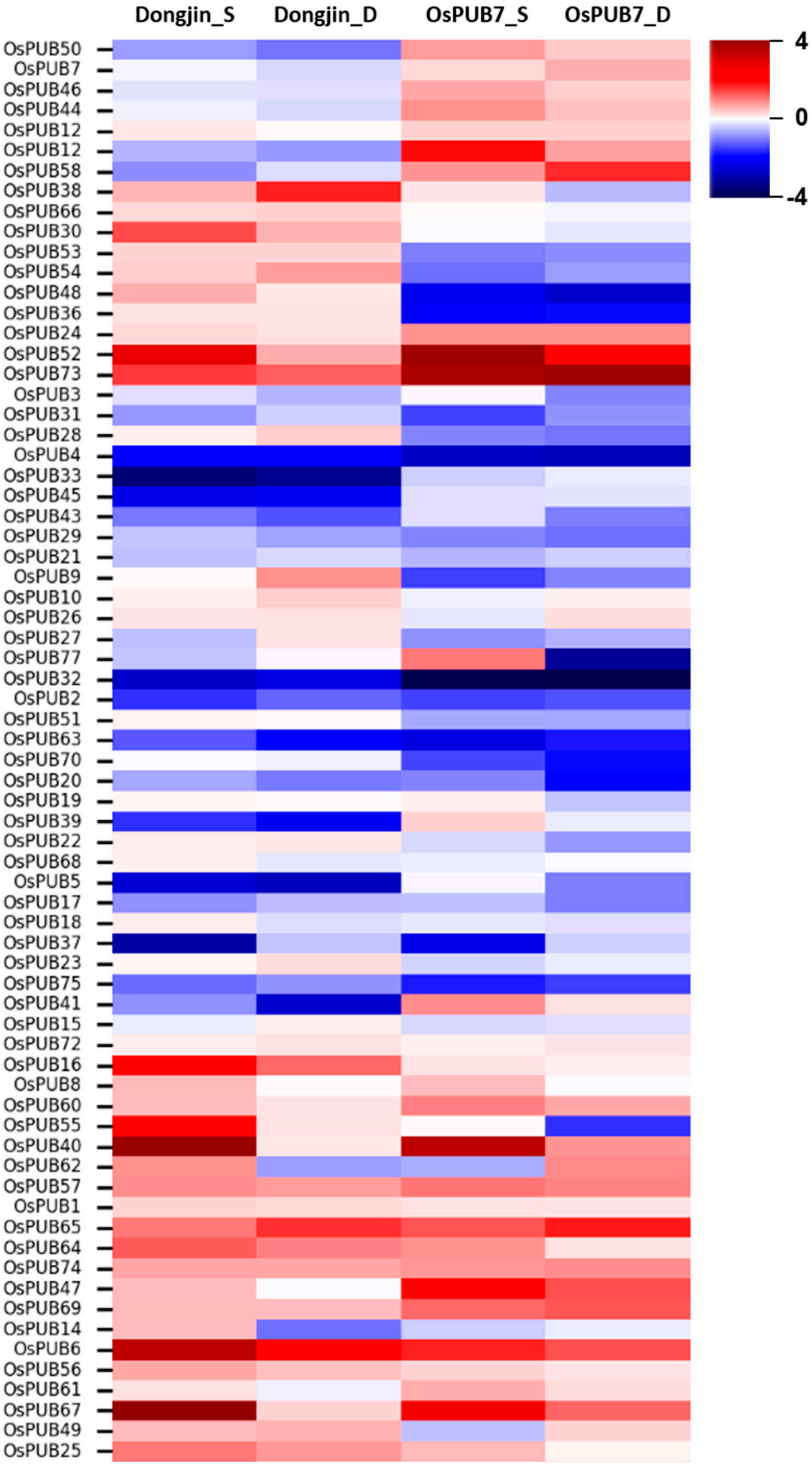


**Supplementary Figure S1.** RNA sequence profiling of OsPUB family genes expression under drought and salinity stress. Heat map displaying expression changes of differentially expressed OsPUB family genes in OsPUB7 gene edited line for drought and salinity stress compared with the control.

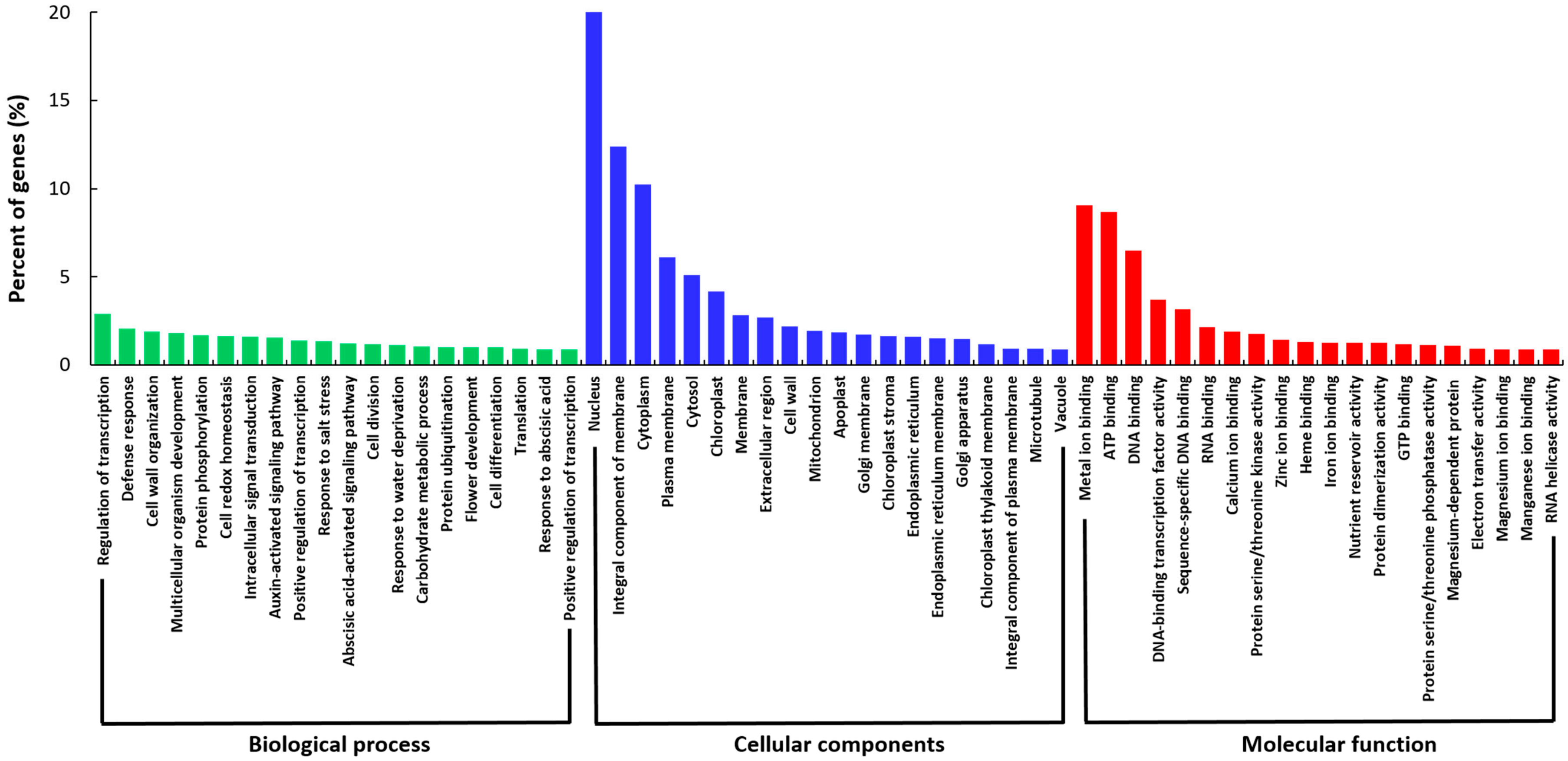


**Supplementary Figure S2.** GO classifications of differentially expressed genes. The differential genes are summarized into three main categories; biological process, cellular components, and molecular function.

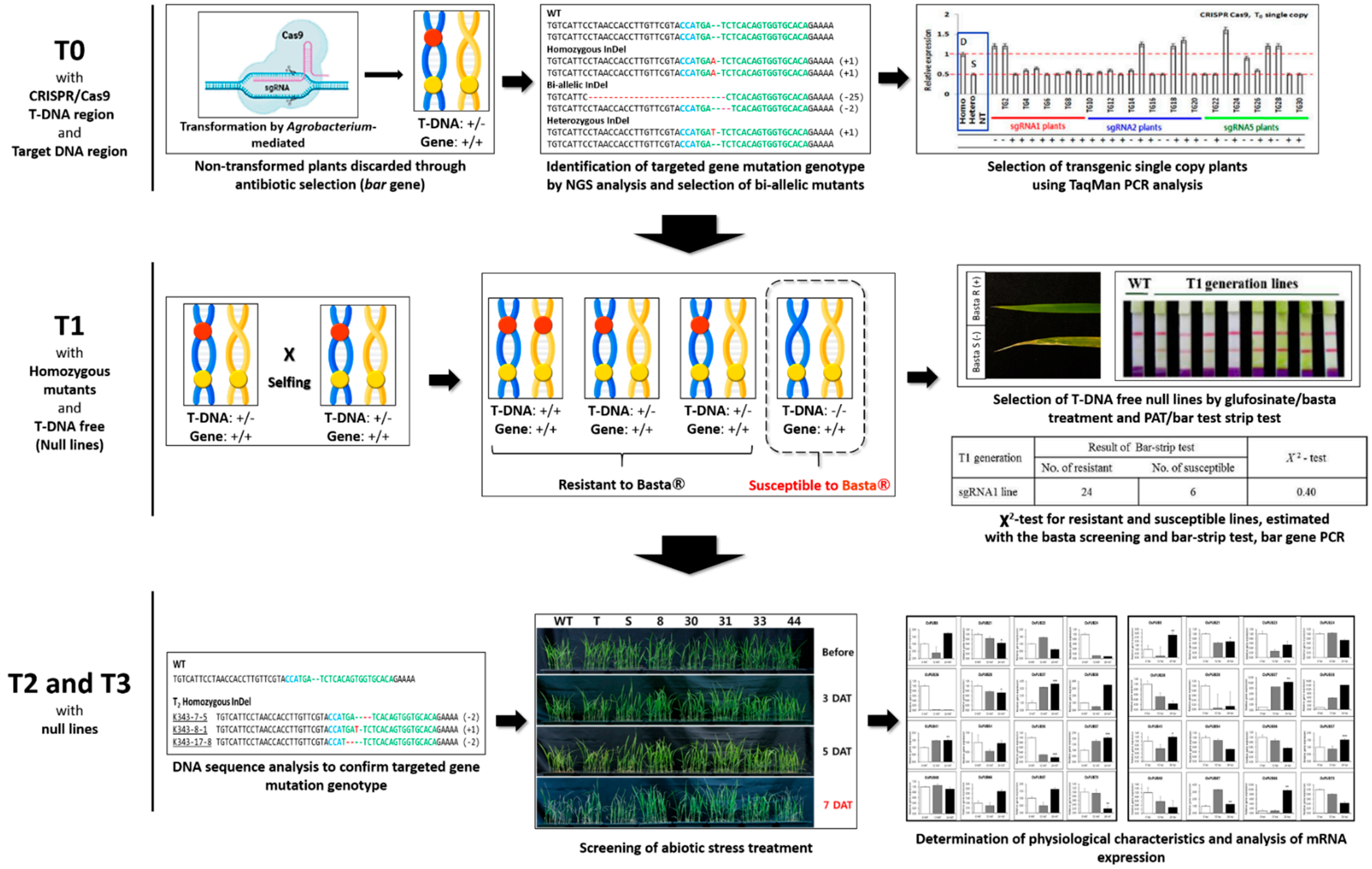


**Supplementary Figure S3.** GO classifications of differentially expressed genes. The differential genes are summarized into three main categories; biological process, cellular components, and molecular function
